# Evaluation of allelopathic effects of *Parthenium hysterophorus* L. methanolic extracts on some selected plants and weeds

**DOI:** 10.1371/journal.pone.0280159

**Published:** 2023-01-06

**Authors:** H. M. Khairul Bashar, Abdul Shukor Juraimi, Muhammad Saiful Ahmad-Hamdani, Md. Kamal Uddin, Norhayu Asib, Md. Parvez Anwar, Ferdoushi Rahaman, Mohammad Amdadul Haque, Akbar Hossain

**Affiliations:** 1 Department of Crop Science, Faculty of Agriculture, University Putra Malaysia, Serdang, Selangor, Malaysia; 2 On-Farm Research Division, Bangladesh Agricultural Research Institute, Gazipur, Bangladesh; 3 Department of Land Management, University Putra Malaysia, Serdang, Selangor, Malaysia; 4 Department of Plant Protection, Faculty of Agriculture, University Putra Malaysia, Serdang, Selangor, Malaysia; 5 Department of Agronomy, Faculty of Agriculture, Bangladesh Agricultural University, Mymensingh, Bangladesh; 6 Department of Agronomy, Bangladesh Wheat and Maize Research Institute, Dinajpur, Bangladesh; Canakkale Onsekiz Mart University, TURKEY

## Abstract

Herbicides made from natural molecules are cost-effective and environmentally friendly alternatives to synthetic chemical herbicides for controlling weeds in the crop field. In this context, an investigation was carried out to ascertain the allelopathic potential of *Parthenium hysterophorus* L. as well as to identify its phenolic components which are responsible for the allelopathic effect. During the observation, the rate of germination and seedlings’ growth of *Vigna subterranea* (L.) Verdc, *Raphanus sativus* (L.) Domin, *Cucurbita maxima* Duchesne., *Cucumis sativus* L., *Solanum lycopersicum* L., *Capsicum frutescens* L., *Zea mays* L., *Abelmoschus esculentus* (L.) Moench, *Daucus carota* L., *Digitaria sanguinalis* (L.) Scop and *Eleusine indica* (L.) Gaertn were investigated by using methanol extracts, isolated from leaf, stem and flower of *P*. *hysterophorus*. Six concentrations (i.e., 25, 50, 75, 100, and 150 g L^-1^) of methanol extracts were isolated from *P*. *hysterophorus* leaf, stem and flower were compared to the control (distilled water). It was also observed that the concentration of methanol extracts (isolated from *P*. *hysterophorus* leaf, stem, and flower) while increased, the rate of seed germination and seedling growth of both selected crops and weeds decreased drastically, indicating that these methanol extracts have allelopathic potential. The allelopathic potential of *P*. *hysterophorus* leaf extraction (811) was found higher than the extraction of the stem (1554) and flower (1109), which is confirmed by EC_50_ values. The principal component analysis (PCA) was also used to re-validate the allelopathic potentiality of these methanol extracts and confirmed that *Raphanus sativus*, *Solanum lycopersicum*, *Capsicum frutescens*, *Abelmoschus esculentus*, *Daucus carota*, *Digitaria sanguinalis*, and *Eleusine indica* were highly susceptible to allelochemicals of *P*. *hysterophorus*. Besides these, the LC-MS analysis also revealed that the *P*. *hysterophorus* leaf extract contained 7 phenolic compounds which were responsible for the inhibition of tested crops and weeds through allelopathic effect. The results of the current study revealed that the leaf of *P*. *hysterophorus* is a major source of allelopathic potential on crops and weeds and which could be used as a valuable natural herbicide in the future for the sustainability of crop production through controlling weeds.

## 1. Introduction

*Parthenium hysterophorus* (L.) is a noxious herb that has now invaded 46 countries and extended its spread from a few islands to around the world, there have been eleven minor and eight major introductions [1]. Its high invasiveness is associated with several factors, including a higher number of seeds production, highly competitive and rapidly expanding, biological plasticity of the life cycle, allelopathic ability and high survival ability against biotic and abiotic stresses [2–5].

*P*. *hysterophorus* is a species of flowering plant in the family Asteraceae. Originally, it was found in the Gulf of Mexico, the USA, the West Indies, and Central America [6]. It is a fast-maturing annual weed with a deep taproot and an erect stem that becomes woody with age. Due to its allelopathic influence, this weed is thought to cause allergic respiratory problems, mutagenicity in humans and livestock, and severe reductions in crop production [7]. Its an annual herbaceous plant that reproduces mostly through seeds. After sprouting, the young plant produces a basal rosette of finely lobed, bright green leaves that are 8–20 cm long and 4–8 cm wide. The rosette stage can continue to grow under unfavourable circumstances and reach a maximum length of 2.5 m [8]. The flower heads are terminal and somewhat hairy; they consist of several small white capitula-shaped florets. Usually, each head has five productive ray florets, although occasionally six or eight. Thousands of branches, which develop in separate clusters, produce compressed black seeds about 2 mm in size [9].

Allelopathy is described as chemicals’ positive or negative impacts on substances formed primarily by plant, microbe, and fungal secondary metabolism on the growth and establishment of neighbouring plants or microorganisms, as well as the dynamical processes of agricultural and natural ecosystems [10]. It’s a complicated phenomenon that’s influenced by a variety of internal and external circumstances. Due to its intricacy, the explanation is a difficult endeavour that necessitates knowledge from a variety of professions [11]. Allelochemicals are plants that release secondary metabolites into the environment. They are anti-inflammatory substances that belong to a variety of chemical classes, primarily phenolic compounds and terpenoids [12]. A comprehensive summary of allelochemicals’ that affects plant growth and development was also observed by Bhadoria [13]. All plant organs (stems, leaves, rhizomes, roots, flowers, pollen, fruits, and seeds) contain allelochemicals, which are released through volatilization, leaf leaching, plant material breakdown, and root exudation. In some way, membrane stability, cell division, elongation, shape and permeability, enzyme activity, and respiration of plants are all influenced. Photosynthesis, protein synthesis, nucleic acid metabolism, and other direct and indirect ways of action cause seed sprouting suppression and limited seedling development [12]. In addition, the microbial breakdown of soil allelochemicals has an impact on the effective dose of allelochemicals that can inhibit plants [14, 15].

Herbicides have been the least expensive and principal method of weed control in developing countries for about 50 years [16]. Herbicides, on the other hand, pose significant risks to agriculture, human health, and the environment. However, increasing crop production without using chemical herbicides is an urgent challenge in crop production. Manual weed management is the most effective and long-term solution for weed management. So, accurate weed control is necessary for food security throughout the world. Therefore, researchers are motivated to seek alternatives because of the labour movement from agriculture to others, and weed biotypes resistant to traditional synthetic pesticides [17]. This strategy will aid in reducing reliance on chemical herbicides, reducing the likelihood of weed resistance to herbicides, reducing health risks and environmental damage, and strengthening the national economy. In the meantime, there are a variety of possible allelochemicals in aerial sections (e.g. leaves) of *Parthenium* weed have been confirmed by several earlier studies; among them p-anisic acid (C_8_H_8_O_3_), p-coumaric acid (C_9_H_8_O_3_), caffeic acid (C_9_H_8_O_4_), ferulic acid (C_4_H_4_O_4_), fumaric acid (C_4_H_4_O_4_), p-hydroxybenzoic acid (C_7_H_6_O_3_), neochlorogenic acid (C_16_H_18_O_9_), protocatechuic acid (C_7_H_6_O_4_), aerulic acid, chlorogenic acid (C_16_H_18_O_9_) and vanillic acid (C_4_H_4_O_4_) are the most important [18, 19] sprouting and development of a plant species in abundance, natural plants are included and different crops and pasture species can be inhibited by these chemicals [20]. Wheat, maize and horse gram [5], lentil [21, 22] and other field crops. Hassan *et al*. [23] showed an inhibitory impact when exposed to parthenium extract. Dhawan and Gupta [19] reported that the extraction of diverse active phytochemicals with flavonoid concentrations works best using methanol as an extraction solvent.

However, there is insufficient evidence of the allelopathic potential of Parthenium methanolic extracts on the sprouting and seedlings development of several crops, particularly bambara groundnut weeds. The bambara groundnut is a new crop for Malaysia, but there is information lacking on the suppression of allelopathy on Bambara groundnut weeds by different parts of *P*. *hysterophorus*. The current study aimed to find out the allelopathic capacity of Parthenium in a laboratory experiment to evaluate the allelopathic suppression of weeds by *P*. *hysterophorus* in Bambara groundnut weeds. The research was directed with the following objectives (1) to evaluate the Allelopathic potential of methanol extracts made from the aerial portions of *P*. *hysterophorus* on target species to develop bioherbicides based on natural products (2) LC-MS was used to identify its phenolic derivatives.

## 2. Materials and methods

### 2.1. Experimental location

Growth chamber research was carried out at Weed Science Lab in the Crop Science Department, Faculty of Agriculture, Universiti Putra Malaysia (3°02’ N, 101°42’ E, elevation 31 m), Malaysia. The temperature in the growth chamber was maintained at 25°C throughout the experimental period.

### 2.2. Experimental treatments and design

Leaf, stem, and floral parts of parthenium was applied at different concentration viz., 0, 25, 50, 75, 100, and 150 g L^-1^ [24]. All treatments were arranged in a completely randomized design (CRD) and repeated four times.

### 2.3. Plant materials and preparation of seeds

For extraction of the leaf of *P*. *hysterophorus* plants, plant materials were taken from Ladang Infoternak farm in Sungai Siput, Perak, Malaysia, and also grown in the net house of field 15 at the University of Putra Malaysia, Selangor, Malaysia. The above-ground part of the plants (excluding roots) were collected just before maturity, rinsed several times using tap water to eliminate dust elements, and then air-dried at ambient temperature (24–26°C) for three weeks. The leaves, stems, and flowers were divided and bulked up into three main parts. In a laboratory blender, both bulked plant components were ground into fine dust and sieved through a 40-mesh sieve.

The inhibitory action of *P*. *hysterophorus* was investigated on nine plant species. Bambara groundnut (*Vigna subterranea* L. Verdc), radish (*Raphanus sativus* L. Domin), sweet gourd (*Cucurbita maxima* Duchesne), tomato (*Solanum lycopersicum* L.), cucumber (*Cucumis sativus* L.), pepper (*Capsicum frutescens* L.), maize (*Zea mays* L.), carrot (*Daucus carota* L.) and okra (*Abelmoschus esculentus* L. Moench) and two weed species goosegrass (*Eleusine indica* L. Gaertn)] and [crab grass (*Digitaria sanguinalis* L. Scop). Crop seeds were attained from Sin Seng Huat Seeds Sdn Bhd Company in Malaysia, while seeds of grasses were personally picked from the Universiti Putra Malaysia’s agricultural field. The seeds were cleaned, air-dried, and stored in airtight containers maintain at –18° C. The vegetable crops are chosen for the determination of ecological effects of allelopathic substances as they represented commonly used species in the field that are recommended by US EPA [25]. They belong to different plant families and can provide great genetic diversity. The seeds germinated 86–95% of the time, according to a random test.

### 2.4. Extract preparation

The extracts were made according to the procedure published by [23, 26]. Accurately 100 g powder from leaves, stems, and flowers of parthenium was placed in a conical flask and allowed to soak in 1L of 80% (v/v) methanol separately. After that, the conical flask was wrapped in paraffin and shaken for 48 hours at 24–26°C room temperatures in an Orbital shaker at 150 rpm agitation speed. To remove debris, cheesecloth in four layers was used to filter the mixtures and centrifuged for one hour at 3000 rpm in a centrifuge (5804/5804 R, Eppendorf, Germany). A single layer of Whatman No. 42 filter paper was used to filter the supernatant. A 0.2-mm Nalgene filter was used to filter the solutions once more to avoid microbial development (Lincoln Park, NJ-based Becton Dickinson percent Labware). Using a rotary evaporator (R 124, Buchi Rotary Evaporator, Germany), the solvents were evaporated from the extract to dryness (a thick mass of coagulated liquid) under vacuum at 40°C and the sample was then collected. From a 100 g sample of *P*. *hysterophorus* powder, the average extracted sample was 17.56 g, which was estimated as per the following formula [1].


[Extractweight(g)/powderweight(g)]×100=Extractionpercentage (1)

For the bioassay, each stock extract from *P*. *hysterophorus* leaves, stems and flowers were diluted in sterile distilled water to provide extract concentrations of 25, 50, 75, 100, and 150 g L^-1^, while purified water was served as control. All extracts were stored at 4° C in the dark until use.

For LC-MS analysis, 100% HPLC GRADE methanol (20 mL) was diluted with the crude sample (20 mg) and filtered through 15-mm, 0.2-μm syringe filters (Phenex, Non-sterile, Luer/Slip, LT Resources Malaysia). Six phenolic derivatives were isolated from methanol extract of *Parthenium hysterophorus* in different parts through LC-MS analysis which has been discussed in detail in Table 1.

**Table 1:** Phenolic derivatives found from methanol extract of *Parthenium hysterophorus* in different parts through LC-MS analysis.

Sl No.	Compound Name	Synonyms	Chemical Formula	Biological activity	Plant part			References
					Leaf	Stem	Flower	
1.	Caffeic acid	3-4-Dihydroxy cinnamic acid	C_9_H_8_O_4_	Antifungal, dermatitis, autotoxic, inhibitory effect to other plants	+	-	+	[27–30]
		3-(3,4-dihydroxy phenyl) acrylic acid						
2.	Ferulic acid	Trans-ferulic acid	C_10_H_10_O_4_		+	-	+	
		4-hydroxy-3-methoxy cinnamic acid						
		Coniferic acid						
		2 Propenoic acid, 3-(4-hydroxy-3-methoxy phenyl)						
3.	Vanillic acid	4-hydroxy-3-methoxybenzoic acid	C_8_H_8_O_4_		+	+	+	
		Benzoic acid, 4-hydroxy-3-methoxy						
4.	Quinic acid	D-(-)-Quinic acid	C_7_H_12_O_6_		+	-	+	
		Chinic acid						
		Quinate						
		1,3,4,5-tetrahydroxy cyclohexanecarboxylic acid						
5.	Parthenin	10-alpha-H-Ambrosa-2,11(13)-1,6-beta di-hydroxy-4-oxo-,gamma–lactone	C_15_H_18_O_4_		+	+	+	
6.	Chlorogenic acid	3,0-caffeoylquinic acid	C_16_H_18_O_9_		+		+	
		3-(3,4-dihydroxy cinnamoyl) quinic acid						
		3-caffeoylquinic acid						
		1,3,4,5-tetrahydroxy cyclohexanecarboxylic acid						
7.	Anisic acid	4-methoxy benzoic acid	C_8_H_8_O_3_		+		+	
		p-anisic acid						
		p-methoxybenzoic acid						

Note: + = present, - = Absent.

### 2.5. Germination and growth bioassays

Healthy, uniform seeds were gathered and treated with 0.2% potassium nitrate for 24 hours (KNO_3_) before being rinsed with distilled water. Twenty Bambara groundnut and sweet gourd seeds and thirty seeds of radish, cucumber, tomato, pepper, maize, okra, carrot, crabgrass, and goosegrass were set up in a sterilized Petri dish with Whatman No. 1 filter paper (90×15 mm). 10 mL of extract of each concentration (25, 50, 75, 100 and 150 g L^-1^) was delivered in Petri dishes, distilled water serving as a control. In a growth chamber, all Petri dishes were inserted. and incubated at 30°C/20°C (day/night) temperature under fluorescent light (8500 lux) on photoperiod 12 h day/12 h night maintaining 30–50% relative humidity. To facilitate gas exchange, the petri dish lids were not sealed.

### 2.6. Identification of phenolic derivatives in *P*. *hysterophorus* leaves, stems, and flowers extracted in methanol

The LC-MS was used to identify the chemical contents of the extracts. The phytochemical compounds of the methanol extracts were performed using LC-MS followed by [31]. LC-MS analysis was performed using Agilent spectrometry equipped with a binary pump. The LC-MS was interfaced with Agilent 1290 Infinity LC system coupled to Agilent 6520 accurate-mass Q-TOF mass spectrometer with a dual ESI source. Full-scan mode from m/z 50 to 500 was performed with a source temperature of 125°C.

The column of Agilent zorbax eclipse XDB-C18, narrow-bore 2.1x150 mm, 3.5 microns (P/N: 930990–902) was used with the temperature 30°C for the analysis. A- 0.1% formic acid in water and B -0.1% formic acid in methanol were used as solvents. Isocratic elution was used to supply solvents at a total flow rate of 0.1 mL minutes^-1^. MS spectra were collected in both positive and negative ion modes. The drying gas was 300°C, with a 10L min-1 gas flow rate and a 45-psi nebulizing pressure. Before analysis, 1 ml of concentration. sample extracts were diluted with methanol and filtered through a 0.22 m nylon filter. The extracts were injected into the analytical column in 1 μl volume for analysis. The mass fragmentations were discovered using an Agilent mass hunter qualitative analysis B.07.00 (Metabolom-ics-2019.m) tool and a spectrum database for organic chemicals.

### 2.7. Data collection

The germination percentage, radicle, and hypocotyl length were measured with a ruler at seven days after seeding. The radicle and hypocotyl length was assessed by software Image J [32] while the inhibition (%) of *P*. *hysterophorus* extracts on a radicle, and hypocotyl length was computed following the formula used by Kordali [33]:

100(C‐A)/C=I (2)

Here, “I” is the percentage of inhibition, “C” control’s mean growth and development and “A” is the aqueous extracts’ mean growth and development.

### 2.8. Statistical analysis

On pooled (two seasons) data, a one-way analysis of variance (ANOVA) was used to regulate any significant variances among concentrations and control. To calculate the difference between the concentration means, the Tukey test (SAS 9.4) with a 0.05 probability level was utilised. EC_r50_, EC_g50_, and EC_h50_ were used to compute real dosages accomplished of suppressing 50% of germination, radicle development, and hypocotyl growth. Based on the suppression of germination (percentage), radicle, and hypocotyl development, Probit analysis was used to compute the EC_g50_, EC_r50_, and EC_h50_ values. From each tested plant, a rank was determined by using the following equation to calculate an index (R_e_) for each of the most active extracts and plants that are the most susceptible:

$$EC_{g50n}\;(germination)+EC_{h50n}\;(hypocotyl)+EC_{r50n}\;(radicle)=Rank\;(R_{e})$$ (3)

Where Re is the plant’s rank n, EC_r50n_, EC_h50n_ and EC_g50n_ are the amounts of plant extract n that inhibit 50% germination, radicle, and hypocotyl length, respectively. The lowest Re value had the maximum active tissue extracts and the utmost sensitive plants, while the highest Re value had the least allelopathic effect of the extract.

The most common application of NTSYSpc 2.02e (Numerical Taxonomy and Multivariate Analysis System) is to do various types of agglomerative cluster analysis of some type of similarity or dissimilarity matrix and the quantity of extract sensitivity among the plants under investigation [34, 35]. The principal component analysis (PCA) was used to re-validate Johnson’s cluster analysis [36].

## 3. Results

### 3.1. Inhibitory influence of *P*. *hysterophorus* on crop species

**Fig 1:**
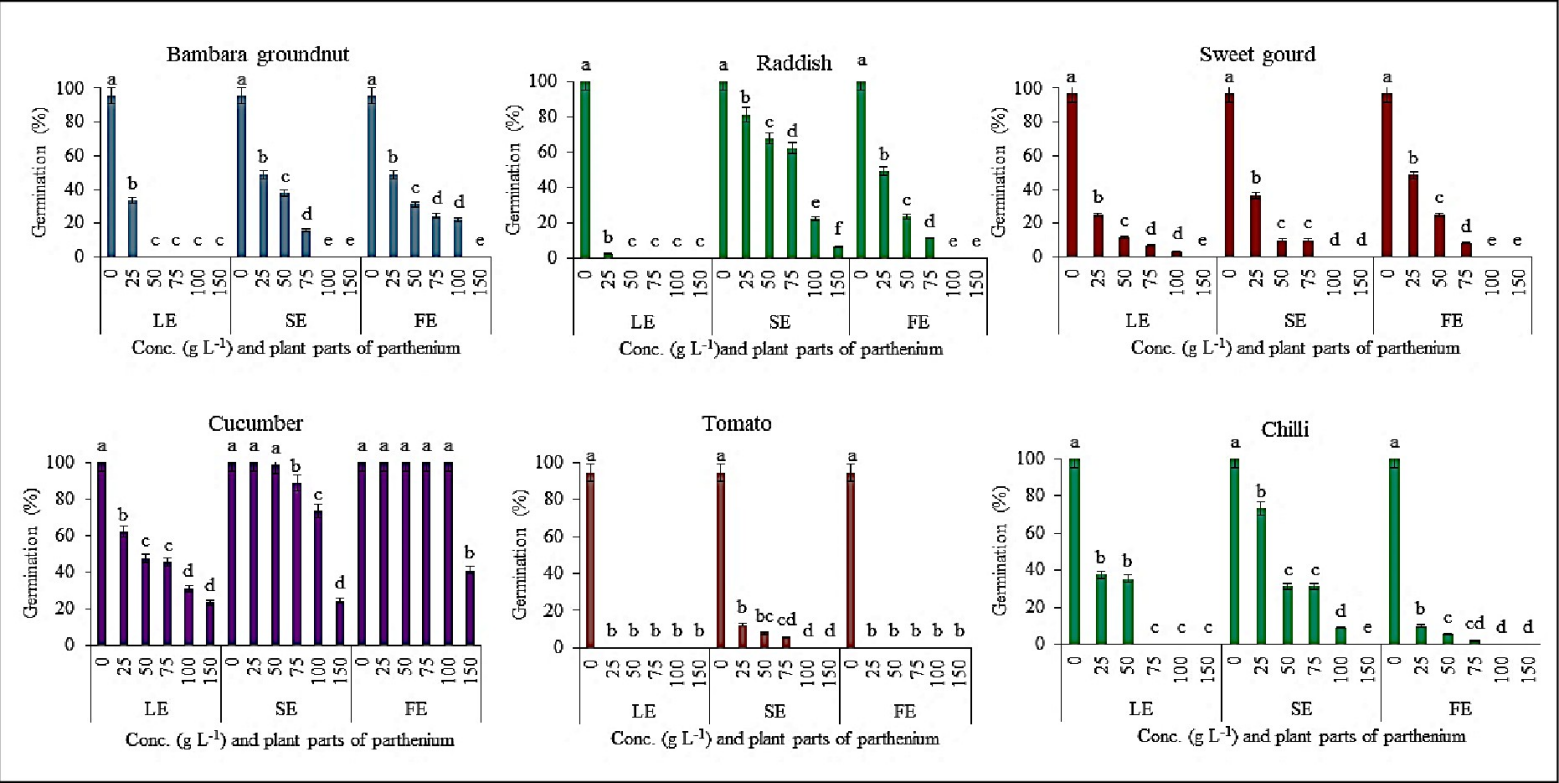
Showing the effect on germination% from different concentration levels of Parthenium aerial plant parts on crops (Bambara groundnut, radish, sweet gourd, cucumber, tomato and pepper). LE- Leaf extract, SE–Stem extract, FE–Flower extract.

Different concentrations of methanolic extracts in the control, Parthenium leaf, stem, and flower concentrations and different crops had a significant influence on the germination of seed, radicle, and hypocotyl length of the examined plants, as well as a rise in extract concentration. Parthenium extracts had a bit stimulatory impact on seed germination at 25 g L^-1^, but an inhibitory effect was observed at higher dosages (Fig 1).

**Fig 2:**
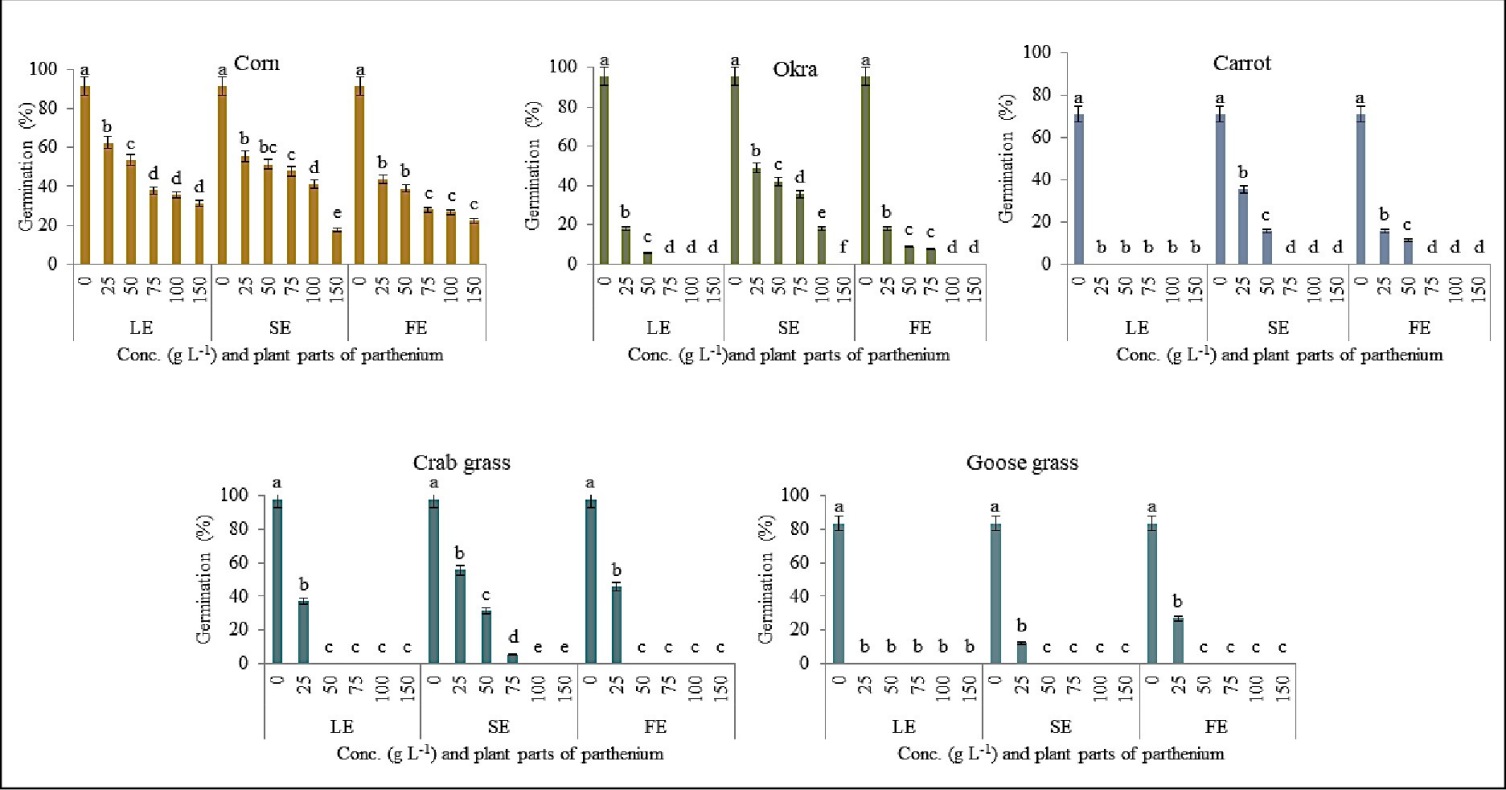
Showing the effect on germination% from different concentration levels of Parthenium aerial plant parts on crops (maize, okra and carrot) and weeds species (crabgrass and goosegrass). LE- Leaf extract, SE–Stem extract, FE–Flower extract.

Methanolic extract of the leaf at 25 g L^-1^ significantly decreased the sprouting of all plants except sweet gourd, cucumber, and maize (p≤0.05), while, seed germination failure was seen in tomato, carrot, and goosegrass if the concentration level further increased. The maximum concentration resulted in 100% germination failure in all crops except cucumber (76%) and maize (65%) (Figs 1 & 2).

When the *P*. *hysterophorus* stem and flower extract were applied at lower doses (25, 50, and 75 g L^-1^), there was no significant reduction in germination (%). When the concentration was raised from 100 to 150 g L^-1^, the sprouting was substantially decreased between 1–100% in the stem and 48–100% in the flower extract among the indicator plants, while it was 61–100% in the leaf extract (Figs 1 & 2). Among them, leaf extract was more affected in many crops than stem and flower extract. On the other hand, germination (%), radicle, and hypocotyl length were significantly decreased at 50 to 100 g L^-1^ leaf extracts (Table 1). Increasing the concentration level eventually reduced the germination percentage over time. Both extracts of Parthenium inhibit the germination percentage of examined indicator for both weed species (Fig 2).

Methanol extracts had a significant allelopathic potential that influenced all the crops on radicle and hypocotyl length studied at varied doses except sweet gourd, cucumber, and maize. Extraction of the leaf at doses more than or equal to 50 g L^-1^ substantially decreased the radicle length of target plants (p≤0.05) (Table 1). With 100 to 150 g L^-1^ stem and flower extract, root development of certain plants was decreased by more than half, whereas the uppermost concentration of the leaf extract (100 to 150 g L^-1^) resulted in no root development such as Bambara groundnut, radish, pepper, okra, etc (Table 1). From the concentration level of 100 to 150 g L^-1^ Parthenium extract radicle length showed the inhibition level of 53–100, 36–100, and 10–100% from leaf, stem, and flower, respectively (Tables 2–4). As a result, the leaf extract had a higher concentration than the others (Table 2).

**Table 2:** Effect of leaves extracts of *Parthenium hysterophorus* with methanol on germination, radicle and hypocotyl length and (%) inhibition of different crops.

Crops	Dose (g L-1)	Leaves extract		
		Inhibition of germination (%)	Length of the radicle (cm)	Length of the hypocotyl (cm)
Bambara groundnut	0	0	1.35±0.05a (0)	0.82±0.01a (0)
	25	65.1	0.72±0.02b (46.7)	0b (100)
	50	100	0c (100)	0b (100)
	75	100	0c (100)	0b (100)
	100	100	0c (100)	0b (100)
	150	100	0c (100)	0b (100)
Radish	0	0	1.23±0.02a (0)	2.30±0.05a (0)
	25	97.7	0.47±0.03b (60.5)	1.20±0.2b (24.5)
	50	100	0c (100)	0c (100)
	75	100	0c (100)	0c (100)
	100	100	0c (100)	0c (100)
	150	100	0c (100)	0c (100)
Sweet gourd	0	0	1.58±0.04a (0)	2.32±0.03a (0)
	25	74.1	0.61±0.02b (61.4)	1.73±0.05b (25.4)
	50	87.9	0.57±0.03b (63.9)	1.64±0.05b (29.3)
	75	93.1	0.37±0.02c (76.6)	1.40±0.08c (39.7)
	100	96.6	0.31±0.03c (80.4)	1.36±0.12c (41.4)
	150	100	0d (100)	0d (100)
Cucumber	0	0	0.91±0.03a (0)	1.80±0.04a (0)
	25	36.4	0.36±0.03b (46.3)	1.35±0.07b (16.7)
	50	51.1	0.31±0.01bc (53.7)	0.89±0.06c (45.1)
	75	53.4	0.28±0.01c (58.2)	0.55±0.03d (66.0)
	100	68.2	0.16±0.01d (76.1)	0.51±0.03de (68.5)
	150	76.1	0.16±0.01d (76.1)	0.40±0.01e (75.3)
Tomato	0	0	0.34±0.01a (0)	0.41±0.01a (0)
	25	100	0b (100)	0b (100)
	50	100	0b (100)	0b (100)
	75	100	0b (100)	0b (100)
	100	100	0b (100)	0b (100)
	150	100	0b (100)	0b (100)
pepper	0	0	0.41±0.02a (0)	-
	25	60.0	0.20±0b (47.4)	-
	50	62.4	0.12±0c (68.4)	-
	75	100	0d (100)	-
	100	100	0d (100)	-
	150	100	0d (100)	-
Maize	0	0	1.98±0.01a (0)	2.73±0.07a (0)
	25	31.7	0.94±0.07b (18.97)	0.85±0.03b (15.84)
	50	41.5	0.68±0.03c (41.38)	0.78±0.03bc (22.77)
	75	58.5	0.64±0.04cd (44.83)	0.77±0.03bc (23.76)
	100	61.0	0.54±0.04de (53.45)	0.73±0.04c (27.72)
	150	65.9	0.42±0.03e (63.79)	0.58±0.02d (42.57)
Okra	0	0	0.70±0.02a (0)	1.36±0.03a (0)
	25	81.4	0.34±0.02b (51.43)	1.08±0.05b (20.59)
	50	94.19	0.25±0.01c (64.29)	0c (100)
	75	100	0d (100)	0c (100)
	100	100	0d (100)	0c (100)
	150	100	0d (100)	0c (100)
Carrot	0	0	0.39±0.01a (0)	0.51±0.01a (0)
	25	100	0b (100)	0b (100)
	50	100	0b (100)	0b (100)
	75	100	0b (100)	0b (100)
	100	100	0b (100)	0b (100)
	150	100	0b (100)	0b (100)
Crabgrass	0	0	0.23±0.02a (0)	0.85±0.03a (0)
	25	62.5	0.10±0b (56.52)	0.22±0.01b (74.12)
	50	100	0c (100)	0c (100)
	75	100	0c (100)	0c (100)
	100	100	0c (100)	0c (100)
	150	100	0c (100)	0c (100)
Goose grass	0	0	0.20±0.02a (0)	0.80±0.01a (0)
	25	100	0b (100)	0b (100)
	50	100	0b (100)	0b (100)
	75	100	0b (100)	0b (100)
	100	100	0b (100)	0b (100)
	150	100	0b (100)	0b (100)

**Table 3:** Effect of stem extracts of *Parthenium hysterophorus* with methanol on germination, radicle and hypocotyl length and (%) inhibition of different crops.

Crops	Dose (g L^-1^)	Stem extract		
		Inhibition of germination (%)	Length of the radicle (cm)	Length of the hypocotyl (cm)
Bambara groundnut	0	0	1.35±0.05a (0)	0.82±0.01a (0)
	25	48.88	1.66±0.03a (1.78)	0b (100)
	50	60.47	1.13±0.02b (33.1)	0b (100)
	75	83.73	0.88±0.04c (47.9)	0b (100)
	100	100	0d (100)	0b (100)
	150	100	0d (100)	0b (100)
Radish	0	0	1.23±0.02a (0)	2.30±0.05a (0)
	25	18.89	1.12±0.02b (8.94)	2.25±0.05a (2.17)
	50	32.23	1.05±0.02c (14.6)	2.19±0.06ab (4.78)
	75	37.78	0.48±0.02d (61.0)	2.07±0.09b (10.0)
	100	77.78	0.30±0.01e (75.6)	0.86±0.05c (62.61)
	150	93.34	0.10±0f (91.9)	0.11±0.01d (95.22)
Sweet gourd	0	0	1.58±0.04a (0)	2.32±0.03a (0)
	25	62.1	1.53±0.03b (17.3)	2.16±0.18a (4.42)
	50	89.7	1.53±0.03b (17.3)	2.09±0.24a (7.52)
	75	89.7	1.60±0.07b (13.51)	1.99±0.15a (11.9)
	100	100	0c (100)	0b (100)
	150	100	0c (100)	0b (100)
Cucumber	0	0	0.91±0.03a (0)	1.80±0.04a (0)
	25	0	0.84±0.05ab (7.69)	1.67±0.01b (7.22)
	50	1.12	0.78±0.05bc (14.29)	1.66±0.02b (7.78)
	75	11.1	0.67±0.04c (26.37)	1.53±0.03c (15.0)
	100	26.7	0.24±0.04d (73.63)	0d (100)
	150	75.6	0.12±0.01d (86.81)	0d (100)
Tomato	0	0	0.34±0.01a (0)	0.41±0.01a (0)
	25	86.25	0.28±0.01b (12.5)	0b (100)
	50	91.26	0.28±0.01b (12.5)	0b (100)
	75	93.76	0.15±0.01c (53.13)	0b (100)
	100	100	0d (100)	0b (100)
	150	100	0d (100)	0b (100)
pepper	0	0	0.41±0.02a (0)	-
	25	26.7	0.29±0.01b (29.27)	-
	50	68.9	0.27±0bc (34.15)	-
	75	68.9	0.26±0.01bc (36.59)	-
	100	91.1	0.25±0.01c (39.02)	-
	150	100	0d (100)	-
Maize	0	0	1.98±0.01a (0)	2.73±0.07a (0)
	25	33.31	1.24±0.06b (21.52)	1.43±0.08a (10.63)
	50	38.66	1.13±0.02c (28.48)	0.94±0.16b (41.25)
	75	42.65	1.11±0.01c (29.75)	0.71b±0.02c (55.63)
	100	50.65	0.63±0.02d (60.13)	0.54±0.02c (66.25)
	150	78.67	0.61±0.03d (61.39)	0.51±0.01c (68.13)
Okra	0	0	0.70±0.02a (0)	1.36±0.03a (0)
	25	45.0	1.19±0.05a (5.56)	2.20±0.1a (4.35)
	50	52.5	1.14±0.06a (9.52)	1.61±0.01b (30.0)
	75	60.0	1.13±0.07a (10.32)	1.44±0.09b (37.39)
	100	80.01	0.80±0.01b (36.51)	0.89±0.03c (61.3)
	150	100	0c (100)	0d (100)
Carrot	0	0	0.39±0.01a (0)	0.51±0.01a (0)
	25	50.0	0.20±0b (48.7)	0b (100)
	50	78.1	0.19±0.01b (51.3)	0b (100)
	75	100	0c (100)	0b (100)
	100	100	0c (100)	0b (100)
	150	100	0c (100)	0b (100)
Crabgrass	0	0	0.23±0.02a (0)	0.85±0.03a (0)
	25	40.48	0.20±0b (66.67)	0.60±0.04b (17.81)
	50	66.68	0.19±0.01bc (68.33)	0.49±0.01c (32.88)
	75	94.05	0.17±0.01c (71.67)	0d (100)
	100	100	0d (100)	0d (100)
	150	100	Od (100)	0d (100)
Goose grass	0	0	0.20±0.02a (0)	0.80±0.01a (0)
	25	83.34	0.14±0.01b (22.22)	0.30±0.01b (46.43)
	50	100	0c (100)	0c (100)
	75	100	0c (100)	0c (100)
	100	100	0c (100)	0c (100)
	150	100	0c (100)	0c (100)

The mean and standard error are used to express the data. The means for each extract with the same letters in the column are not substantially different at p≤0.05. Inhibition percentages relative to the control are shown inside the parenthesis.

**Table 4:** Effect of flower extracts of *Parthenium hysterophorus* with methanol on germination, radicle and hypocotyl length and (%) inhibition of different crops.

Crops	Dose (g L^-1^)	Flower extract			
		Length of the hypocotyl (cm)	Inhibition of germination (%)	Length of the radicle (cm)	Length of the hypocotyl (cm)
Bambara groundnut	0	0.82±0.01a (0)	0	1.35±0.05a (0)	0.82±0.01a (0)
	25	0b (100)	48.8	0.93±0.01b (7.0)	0.90±0.09b (29.1)
	50	0b (100)	67.5	0.93±0.03b (7.0)	0.84±0.08b (33.9)
	75	0b (100)	74.4	0.91±0.01b (9.0)	0.80±0.21b (37.0)
	100	0b (100)	76.7	0.90±0.02b (10.0)	0.21±0.1c (83.5)
	150	0b (100)	100	0c (100)	0d (100)
Radish	0	2.30±0.05a (0)	0	1.23±0.02a (0)	2.30±0.05a (0)
	25	2.25±0.05a (2.17)	51.12	0.56±0.08b (58.8)	1.62±0.03b (42.76)
	50	2.19±0.06ab (4.78)	76.67	0.37±0.02c (72.8)	1.50±0.03c (47.0)
	75	2.07±0.09b (10.0)	88.89	0.30±0.01c (77.9)	0.42±0.02d (85.16)
	100	0.86±0.05c (62.61)	100	0d (100)	0e (100)
	150	0.11±0.01d (95.22)	100	0d (100)	0e(100)
Sweet gourd	0	2.32±0.03a (0)	0	1.58±0.04a (0)	2.32±0.03a (0)
	25	2.16±0.18a (4.42)	49.13	0.67±0.07b (31.63)	1.99±0.07a (3.4)
	50	2.09±0.24a (7.52)	73.68	0.65±0.08bc (33.67)	1.99±0.07a (3.4)
	75	1.99±0.15a (11.9)	91.23	0.51±0.02c (47.96)	1.98±0.1a (3.88)
	100	0b (100)	100	0d (100)	0b (100)
	150	0b (100)	100	0d (100)	0b (100)
Cucumber	0	1.80±0.04a (0)	0	0.91±0.03a (0)	1.80±0.04a (0)
	25	1.67±0.01b (7.22)	0	0.43±0.08b (37.68)	1.95±0.08a (7.58)
	50	1.66±0.02b (7.78)	0	0.26±0.01c (62.32)	1.23±0.06b (41.7)
	75	1.53±0.03c (15.0)	0	0.24±0.02c (65.22)	1.09±0.05b (48.3)
	100	0d (100)	0	0.24±0.02c (65.22)	1.06±0.06b (49.8)
	150	0d (100)	58.9	0.22±0.01c (68.12)	0.61±0.01c (71.1)
Tomato	0	0.41±0.01a (0)	0	0.34±0.01a (0)	0.41±0.01a (0)
	25	0b (100)	100	0b (100)	0a (100)
	50	0b (100)	100	0b (100)	0a (100)
	75	0b (100)	100	0b (100)	0a (100)
	100	0b (100)	100	0b (100)	0a (100)
	150	0b (100)	100	0b (100)	0a (100)
pepper	0	-	0	0.41±0.02a (0)	-
	25	-	89.5	0.28±0.04b (37.78)	-
	50	-	94.2	0.23±0.02bc (48.89)	-
	75	-	97.7	0.18±0c (60.0)	-
	100	-	100	0d (100)	-
	150	-	100	0d (100)	-
Maize	0	2.73±0.07a (0)	0	1.98±0.01a (0)	2.73±0.07a (0)
	25	1.43±0.08a (10.63)	52.44	1.41±0.06b (28.79)	2.60±0.08a (4.76)
	50	0.94±0.16b (41.25)	57.32	1.23±0.02c (37.88)	2.30±0.16b (15.75)
	75	0.71b±0.02c (55.63)	69.52	1.21±0.01cd (38.89)	1.78±0.02c (34.8)
	100	0.54±0.02c (66.25)	70.74	1.13±0.02d (42.93)	1.69±0.02c (38.1)
	150	0.51±0.01c (68.13)	75.61	0.74±0.03e (62.63)	1.20±0.01d (56.04)
Okra	0	1.36±0.03a (0)	0	0.70±0.02a (0)	1.36±0.03a (0)
	25	2.20±0.1a (4.35)	80.73	0.43±0.01b (51.14)	0b (100)
	50	1.61±0.01b (30.0)	90.37	0.41±0.01b (53.41)	0b (100)
	75	1.44±0.09b (37.39)	91.57	0.40±0.01b (54.55)	0b (100)
	100	0.89±0.03c (61.3)	100	0c (100)	0b (100)
	150	0d (100)	100	0c (100)	0b (100)
Carrot	0	0.51±0.01a (0)	0	0.39±0.01a (0)	0.51±0.01a (0)
	25	0b (100)	75.9	0.28±0.05b (20.0)	0.46±0.03b (13.51)
	50	0b (100)	82.8	0.20±0c (42.9)	0c (100)
	75	0b (100)	100	0d (100)	0c (100)
	100	0b (100)	100	0d (100)	0c (100)
	150	0b (100)	100	0d (100)	0c (100)
Crabgrass	0	0.85±0.03a (0)	0	0.23±0.02a (0)	0.85±0.03a (0)
	25	0.60±0.04b (17.81)	51.19	0.10±0b (47.37)	0.26±0b (54.39)
	50	0.49±0.01c (32.88)	100	0c (100)	0c (100)
	75	0d (100)	100	0c (100)	0c (100)
	100	0d (100)	100	0c (100)	0c (100)
	150	0d (100)	100	0c (100)	0c (100)
Goose grass	0	0.80±0.01a (0)	0	0.20±0.02a (0)	0.80±0.01a (0)
	25	0.30±0.01b (46.43)	66.67	0.12±0b (62.5)	0.53±0.01b (46.46)
	50	0c (100)	100	0c (100)	0c (100)
	75	0c (100)	100	0c (100)	0c (100)
	100	0c (100)	100	0c (100)	0c (100)
	150	0c (100)	100	0c (100)	0c (100)

The mean and standard error are used to express the data. The means for each extract with the same letters in the column are not substantially different at p≤0.05. Inhibition percentages relative to the control are shown inside the parenthesis.

Furthermore, we observed that the weed crabgrass and goosegrass were severely affected by leaf and flower methanol extract in the doses of 50 to 150 g L^-1^, but stem extract was affected by doses of 100 to 150 g L^-1^. So, it was observed that severely affected weed by leaf than flower and stem plant parts. The amount of inhibition rose when the concentration level was raised. Different components of Parthenium reduced the shoot length of all examined plants by 27–100, 61–100 and 38–100%, respectively, at the doses of 100 to 150 g L^-1^.

### 3.2. The half inhibitory effect of Parthenium methanol extracts

Table 5 showed the half inhibitory (EC_50_) impact of Parthenium plant parts with methanol extracts, as well as the sensitivity of the evaluated starting growth parameters and plants. The efficacy of stem extract (1554) was lower than that of leaf extract (811), and it was followed by flower extract (1109) in all tested crops. The EC_50_ value showed some differences in sensitivity between the tested plant’s responses to the inhibitory influence of *P*. *hysterophorus* (Table 5). In the case of leaf methanol extract maize, cucumber and sweet gourd was only impacted at higher concentrations. The rank value of these crops is 463, 144, and 108 respectively, which means that these crops are more tolerant, which shows that more doses need to destroy these plants. On the other hand, Bambara groundnut, radish, tomato, carrot, crabgrass, and goosegrass are more sensitive to leaf methanol extract next to pepper (53) and okra (41).

**Table 5:** For the examined species, the rank value (Re) of *P*. *hysterophorus* methanol extract.

Target plants	Leaf extract			
	ECg50	ECr50	ECh50	Rank
	Values in g L^-1^			
Bambara groundnut	0	0	0	0
Radish	0	0	0	0
Sweet gourd	12.98	22.35	73.26	108.59
Cucumber	48.97	35.11	60.07	144.15
Tomato	0	0	0	0
pepper	25.17	28.76	0	53.93
Maize	62.33	85.60	315.38	463.31
Okra	13.76	28.01	0	41.77
Carrot	0	0	0	0
Crabgrass	0	0	0	0
Goosegrass	0	0	0	0
Rank	163.21	199.83	448.71	811.75
**Stem extract**				
Bambara groundnut	30.48	62.42	0	92.9
Radish	64.67	68.91	93.78	227.36
Sweet gourd	19.78	68.16	77.20	165.14
Cucumber	119.62	83.05	74.86	277.53
Tomato	7.09	61.23	0	68.32
pepper	39.82	72.20	0	112.02
Maize	71.28	100.97	72.39	244.64
Okra	38.10	99.18	74.53	211.81
Carrot	26.59	31.04	0	57.63
Crabgrass	31.62	20.79	44.41	96.82
Goosegrass	0	0	0	0
Rank	449.05	667.95	437.17	1554.17
**Flower extract**				
Bambara groundnut	29.20	114.79	56.50	200.49
Radish	26.33	23.86	35.43	85.62
Sweet gourd	27.54	49.26	75.02	151.82
Cucumber	143.50	37.19	83.40	264.09
Tomato	0	0	0	0
pepper	6.46	40.59	0	47.05
Maize	23.31	108.16	126.93	258.4
Okra	10.14	34.24	0	44.38
Carrot	16.04 (0)	41.69	0	57.73
Crabgrass	0	0	0	0
Goosegrass	0	0	0	0
Rank	282.52	449.78	377.28	1109.58

The quantities of extracts that inhibit 50% of germination, root, and hypocotyl, respectively, are designated as EC_g50_, EC_r50_, and EC_h50_.

Again, in the case of stem methanol extract cucumber (277), maize (244), radish (227), okra (211), sweet gourd (165) and pepper (112) are more tolerant and other crops are more sensitive. It was inhibited by the extract. On the contrary, in the case of flower extract cucumber (264), maize (258), Bambara groundnut (200), and sweet gourd (151) are more tolerant than tomato, crabgrass, and goosegrass with other crops are more sensitive. These findings revealed that *P*. *hysterophorys* leaf extract had a greater effect on plant development than flower and stem extract at all dosages. Again, germination was seriously affected (163, 449, and 282) among the leaf, stem, and flower extracts indices, while radicle length (199, 667, and 449) and hypocotyl length (448, 437, and 377) were less affected to both plant sections. Overall, the methanol leaf extract of *P*. *hysterophorus* was very hazardous to all plants examined, particularly to germination, which was hindered at the lowest dosage.

### 3.3. Cluster and Principal Component Analysis (PCA)

**Fig 3:**
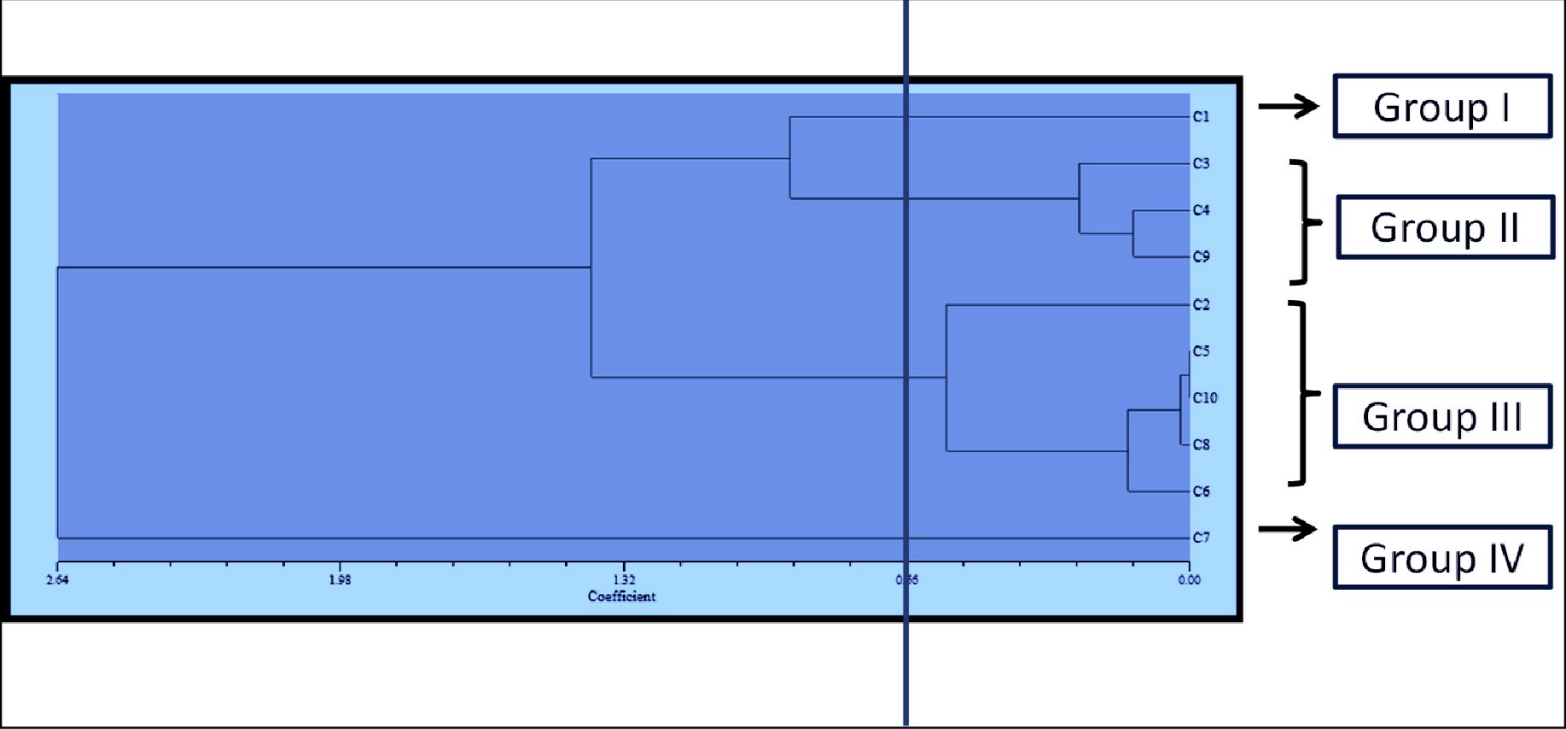
All indicator plants’ mean EC_50_ values for seed germination, radicle, and hypocotyl length are represented in a dendrogram (C_1_- Bambara groundnut, C_2_- Radish, C_3_- Sweet gourd, C_4_- Cucumber, C_5_- Tomato, C_6_- pepper, C_7_- Maize, C_8_- Okra, C_9_- Carrot, C_10_- Crabgrass, C_11_- Goosegrass) treated with the leaf, stem and flower extracts of *P*. *hysterophorus* with methanol revealed by non-overlapping (SAHN) UPGMA method.

Cluster analysis was also used to categorize distinct groups of plants with comparable responses to the inhibition of leaf, stem, and flower extracts by combining all three characteristics examined. Cluster analysis produced a dendrogram that revealed variation in sensitivity among the plants (Fig 3).

Plants may be divided into four classes based on how they react to leaf, stem, and flower extracts (Table 6). According to Table 6, group IV comprises tolerant monocot plants, whereas the dicot plants examined are referred to as the sensitive groups. Maize was recorded tolerant, whereas the moderately sweet gourd, cucumber, and carrot had an intermediate reaction to the allelopathic potential. The most vulnerable plants, on the other side, were Bambara groundnut, radish, tomato, crabgrass, goosegrass, okra, and pepper. Overall, the dicot plants were shown to be more active against the Parthenium extract than the monocots.

**Table 6:** Showing the similarity among the indicator plants.

Clustering	Code	Name of the crop
Group I	C_1_	Bambara groundnut
Group II	C_3_, C_4_, C_9_	Sweet gourd, Cucumber, Carrot
Group III	C_2_, C_5_, C_10_, C_11_, C_8_, C_6_	Radish, Tomato, Crabgrass, Goosegrass, Okra, pepper
Group IV	C_7_	Maize

**Fig 4:**
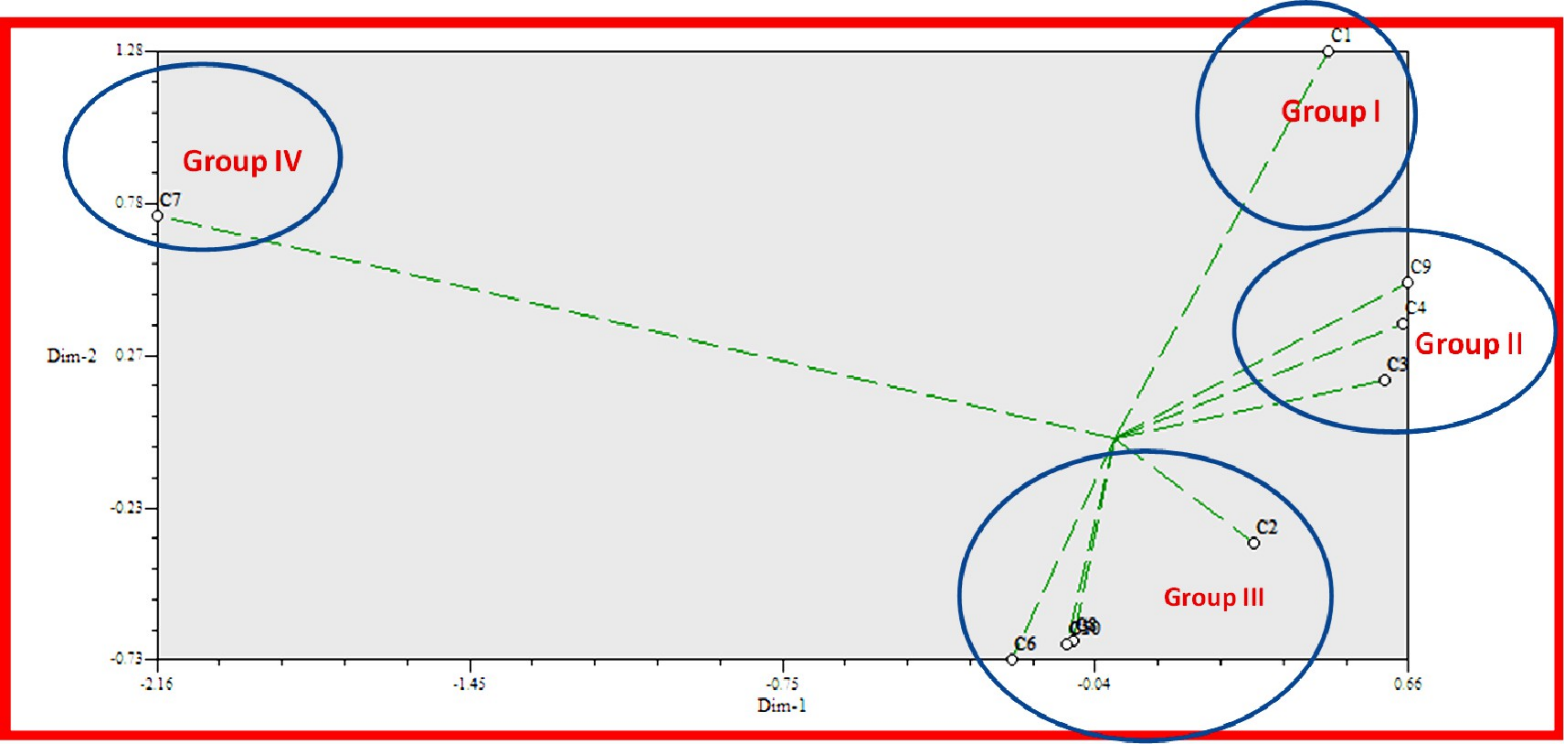
Based on Euclidian distance, the principal component analysis (PCA)-2D graphical association among the indicator plants treated with Parthenium leaf, stem, and flower with methanol extract.

**Fig 5:**
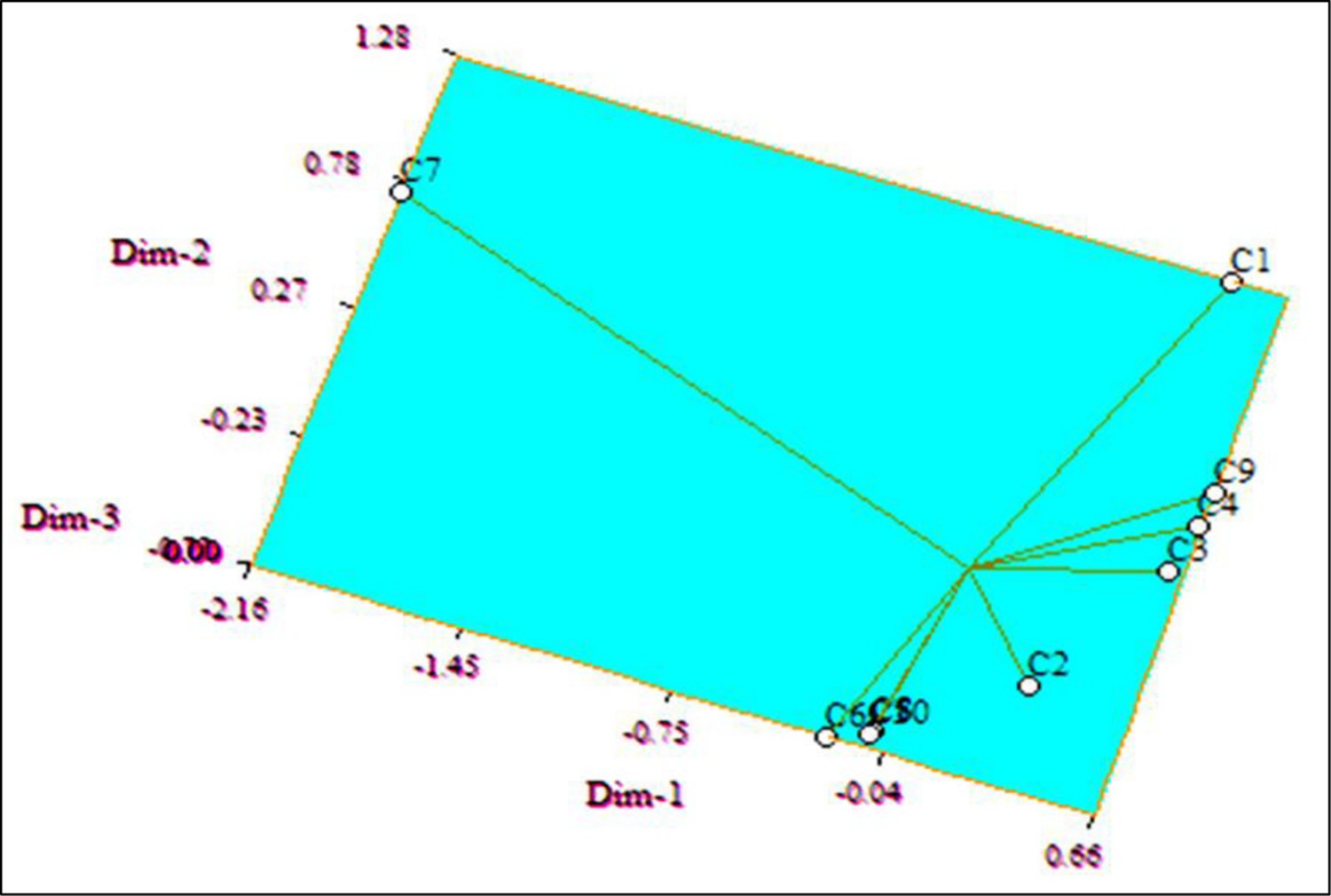
Based on Euclidian distance, principal component analysis (PCA)-3D graphical association between the indicator plants treated with Parthenium leaf, stem, and flower with methanol extract.

The principal component analysis (PCA), on the other hand, is a re-validation tool for cluster analysis. Johnson uses PCA to estimate the total variation that exists in a set of characters. As shown by the eigenvector in the two-dimensional (Fig 4) and three-dimensional (Fig 5) graphical elucidations, the majority of the indicator plants were spread at short distances, while just two were dispersed at long distances. Bambara groundnut and Maize were the accessions that were farthest from the centroid, whilst other accessions were close to it.

### 3.4. Identified phenolic derivatives from LC-MS analysis

**Fig 6:**
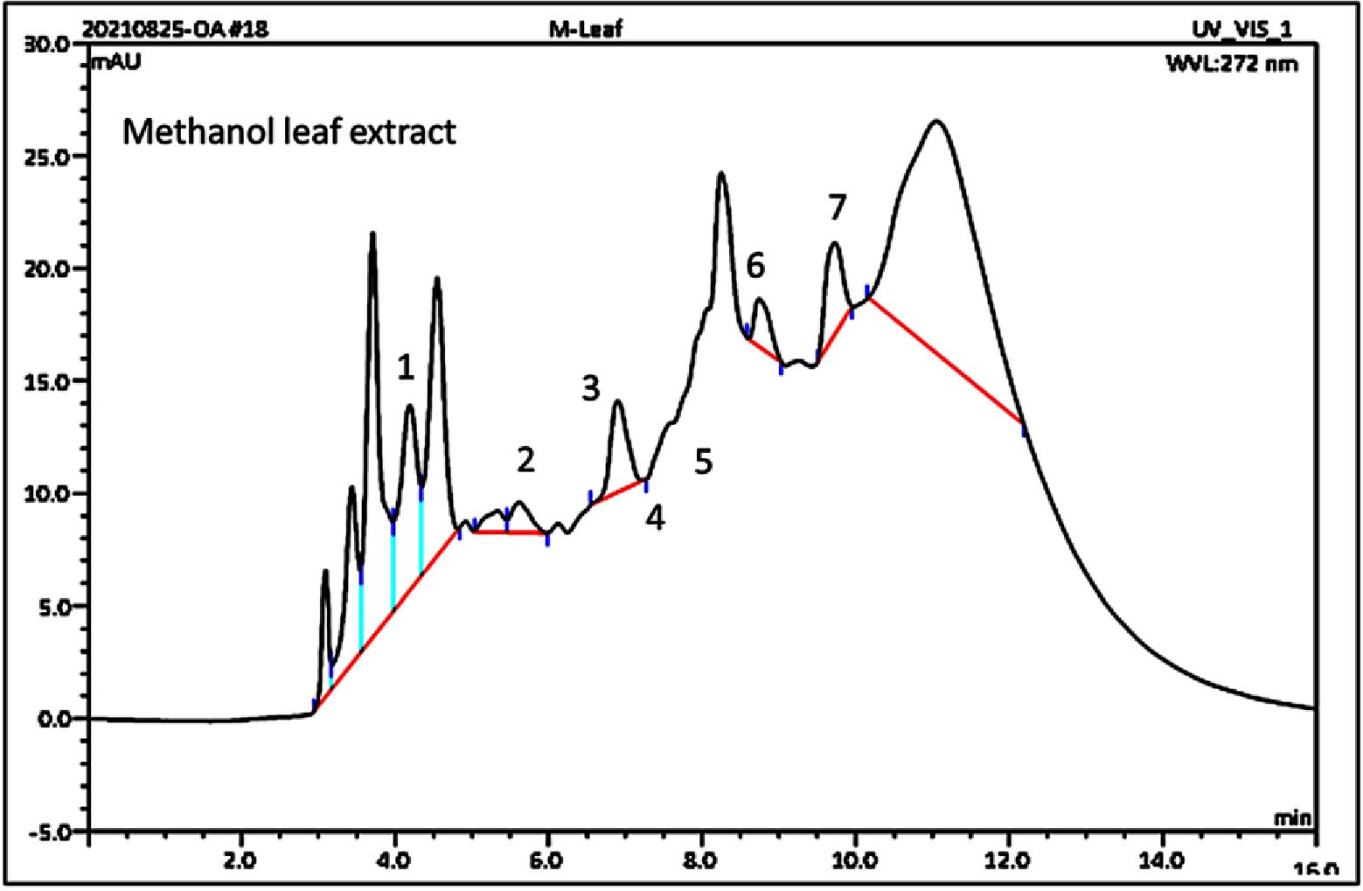
Chromatograms of allelopathic potential of standard compounds from leaf extract of *P*. *hysterophorus* (1. Parthenin, 2. Quinic acid, 3. Chlorogenic acid, 4. Vanillic acid, 5. Caffeic acid, 6. Ferulic acid, and 7. Anisic acid).

The identified phenolic derivatives of *P*. *hysterophorus* plant parts with methanolic extracts through LC-MS analysis are listed in Table 1. The leaf, stem, and flower extracts of *P*. *hysterophorus* have diverse chemical compositions. A total of 7 Phenolic derivatives were detected from methanol extract of *P*. *hysterophorus* in different parts through LC-MS analysis (Table 1; Fig 6). These phenolic derivatives are responsible for inhibition of other plants, autotoxic, and dermatitis. Parthenin and other phenolic acids found in the leaf and flower extracts include vanillic acid, caffeic acid, quinic acid, anisic acid, chlorogenic acid, and ferulic acid, contrary Parthenin, vanillic acid found in the stem extract. The amount and kind of chemicals discovered in each plant were found to be proportional to herbicidal action. As a consequence, the compound of the various plant parts inhibited indicator plant germination and seedling growth, with the extraction of the leaf having a greater inhibitory influence than the other plant parts.

## 4. Discussion

*P*. *hysterophorus* with methanol extract influenced germination (%) and growth of seedlings of nine different crops (*V*. *subterranean*, *R*. *sativus*, *C*. *maxima*, *C*. *sativus*, *S*. *lycopersicum*, *C*. *annum*, *Z*. *mays*, *A*. *esculentus*, *D*. *carota*) and two weed species (*D*. *sanguinalis* and *E*. *indica*). In a dose-dependent way, all portions of Parthenium extracts affected germination, radicle length, and hypocotyl length in the tested species. Because of its exceptional strength, efficacy, and consistency in preventing germination and seedling development, extracts of Parthenium leaf were the most promising. Plant extracts are hypothesized to decrease germination through having osmotic potential on the rate of absorption, which in turn affects germination and, in particular, cell elongation [36].

Wheat, maize, and horse gram seedling growth were inhibited by extracts of *P*. *hysterophorus* methanol extract. Its demonstrated greater inhibitory power, in comparison to the aqueous extract [37]. Dhawan & Gupta [19] reported that the extraction of different active phytochemicals with flavonoid concentration works best-using methanol as an extraction solvent. The germination of *V*. *radiata* seeds was tested for up to 120 hours using methanol crude Parthenium extracts and it was discovered that there is a considerable difference in germination kinetics between the treatments of methanol crudes [38].

Tef germination was significantly reduced at intermediate to higher concentrations when Parthenium flower and leaf extracts were used. This suggests that inhibitory compounds are present in larger concentrations in flower and leaf than in stem and root sections [39, 40]. The fact that roots came into direct touch with the extract and then with inhibitory compounds, as reported in previous research with a variety of crops and weeds [41, 42].

The aerial parts extract of *P*. *hysterophorus* had a substantial influence on the germination of seed, radicle and hypocotyl length reduction in this investigation. These effects grew stronger as the concentration level increased. These discoveries are consistent with those of Wakjira et al. [43] and Mersie and Singh [44] who discovered a robust link between greater *P*. *hysterophorus* aqueous extract concentrations and increased poisonousness to agronomic crops and weeds. The effects of secondary metabolites generated by *P*. *hysterophorus* aerial parts on growth and development in Bambara groundnut weeds and chosen species. Phytochemicals isolated from *P*. *hysterophorus* stems, leaves, and flowers methanol extracts were competent to alter crops and weed seedling sprouting and development. Similarly, Motmainna et al. [45] discovered that *P*. *hysterophorus* extract had a considerable impact on the germination and development of the weed species. The degree of inhibition was raised when the concentration of the extract was increased. Radicle growth is more vulnerable to allelopathic plant extracts than other organs due to radicles being the first tissue to be shown to be phytotoxic chemicals and have a more absorbent tissue than other parts [46, 47], and/or the root apical meristem has a low mitotic division rate [48]. Furthermore, allelopathic elements can suppress the production of radicle and epidermis by altering genes involved in cellular characterization [49]. Parthenium extract was more effective than the *B*. *alata* and *C*. *rutidosperma* extract [45]. This is inconsistent with [50], who discovered that extracts of the allelopathic plant have a stronger effect on radicle length than hypocotyl development. This could be due to the roots being the initial ones to attract allelochemical substances from the atmosphere.

The survivability rate of the target plants was inhibited by varying doses of *P*. *hysterophorus* leaf, stem, and flower methanol extracts. Maximum doses of methanol extracts included more inhibitory chemicals, resulting in more inhibition. In the same way, Han et al. [51] reported that the allelopathic potential of *P*. *hysterophorus* extracts was concentration-dependent, and allelopathic potential rose as extract concentration was raised. It was also claimed that the leaf extract had a more inhibitory allelopathic activity than other vegetative portions, and phytochemical research had already revealed a larger accumulation of growth inhibitors in *P*. *hysterophorus* leaves [52]. At all doses examined, the extracts inhibited *P*. *minor* germination, and when extract concentrations increased then inhibition increased [35]. However, extracts from the leaves had a higher level of toxicity than extracts from the stem [53].

Different plant species’ susceptibility to inhibitory chemicals has been documented for a variety of causes. Msafiri et al. [54] observed that both tested species showed substantial allelopathic effects of *P*. *hysterophorus* seed and leaf aqueous extract on seed sprouting, root and hypocotyl length, and fresh and dry mass. According to Kobayasi [55], each species’ have biological characteristics. The seed structure and seed coat penetrability can also play a role in different reactions to similar allelopathic extracts [56]. Higher concentration reduced the seedling length of all the test crops but, sweet gourd, maize, and cucumber were less sensitive than other crops. This may be due to genotypic variation in response to the higher concentration of extracts. Similar results of the inhibitory effect were observed by Aslani et al. [57]. These findings are confirmed by the authors’ other findings who stated that phytotoxic compounds are more vulnerable to smaller plants, although phytotoxin reactions differed from species to species [58].

The phytochemical screening revealed a huge number of compounds in the *P*. *hysterophorus* extracts, some of which have previously been identified as poisons in several investigations [59, 60]. Furthermore, various plant sections of *P*. *hysterophorus* contained a different number of compounds. The quantity of toxic compounds was more in the leaf than in the other plant parts; as a result, the leaves have a stronger inhibitory effect. *P*. *hysterophorus* leaves release allelochemicals into the soil by leaching or decomposition, and have the potential to impair the development of other plants by altering the physicochemical properties of soil, according to Dogra & Sood [61]. Arowosegbe & Afolayan [62] also found that beetroot (*Beta vulgaris* L.), Turnip (*Brassica rapa* L.), and carrot (*D*. *carota* L.) were all inhibited more by *Aloe ferox* Mill. leaf than by the root extract. The suppressive influence of extracts, according to Verdeguer et al. [63] is determined by the extract’s chemical makeup as well as the plant sections to which it is applied. These findings are consistent with those of Javaid and Anjum [64] and Verma et al. [65] who discovered that parthenin and other phenolic acids such as caffeic acid, vanillic acid, anisic acid, chlorogenic acid, and para hydroxybenzoic acid are the most responsible for plant growth inhibition.

## 5. Conclusions

The results and discussion of the study revealed that herbicides made from natural molecules are cost-effective and environmentally friendly alternatives to synthetic chemical herbicides for controlling weeds in the crop field. To fulfil the objectives of the study, six concentrations (i.e., 25, 50, 75, 100, and 150 g L^-1^) of methanol extracts were isolated from *P*. *hysterophorus* leaf, stem and flower and were compared to the control (distilled water). The concentration of methanol extracts (isolated from *P*. *hysterophorus* leaf, stem, and flower); while increased, the rate of seed germination and seedling growth of *Vigna subterranea* (L.) Verdc, *Raphanus sativus* (L.) Domin, *Cucurbita maxima* Duchesne., *Cucumis sativus* L., *Solanum lycopersicum* L., *Capsicum frutescens* L., *Zea mays* L., *Abelmoschus esculentus* (L.) Moench, *Daucus carota* L., *Digitaria sanguinalis* (L.) Scop and *Eleusine indica* (L.) Gaertn decreased drastically, indicating that these methanol extracts have allelopathic potential. The allelopathic potentiality leaf extract of *P*. *hysterophorus* was found higher than the extraction of the stem (1554) and flower (1109), which is confirmed by EC_50_ values, the principal component analysis (PCA) and LC-MS analysis, it is due to the leaf extract of *P*. *hysterophorus* contained 7 phenolic compounds which were responsible for inhibition of tested crops and weeds. Therefore, it can be concluded that the leaf phenolic compounds of *P*. *hysterophorus* may be used as a valuable natural herbicide in the future for the sustainability of crop production by controlling weeds.
